# 

**Published:** 1877-04

**Authors:** 


CATALOGUE OF EXCHANGES.—American Clinical lectures, New York; American Journal of Microscopy, New York; American Journal of the Medical Sciences, Philadelphia; American Journal of Pharmacy, Philadelphia; American Medical Bi-Weekly, Louisville, Ky.; American Practitioner, Indianapolis, Indiana; Archives of Clinical Surgery, New York; Archives of Dermatology, New York; Baltimore Physician and Surgeon, Baltimore, Md.; Boston Journal of Chemistry, Boston, Mass.; Boston Medical and Surgical Journal, Boston, Mass.; Braithwait's Retrospect, New York; Buffalo Medical and Surgical Journal, Buffalo, New York; Canada Journal of Medical Sciences, Toronto, Canada; Cincinnati Lancet and Observer, Cincinnati, O.; Detroit medical Journal, Detroit, Mich.; Druggists' Advertiser, New York; Druggists' Circular, New York; Half Yearly Compendium of Medical Sciences, Philadelphia; Herald of Health, New York; Journal of Materia Medica, New Lebanon, N. Y.; Journal of Nervous and Mental Diseases, Chicago, Ill.; London Lancet, New York; Louisville Medical News, Louisville, Ky.; Medical and Surgical Reporter, Philadelphia, Pa.; Medical Brief, St. Louis, Mo.; Medical Eclectic, New York; Medical Intelligencer, Philadelphia, Pa.; Medical Journal and Examiner, Chicago, Ill.; Medical News and Library, Philadelphia, Penn.; Medical Record, New York; Metric Bulletin, Boston, Mass.; Monthly Abstract of Medical Science; Philadelphia, Pa.; Nashville Journal of Medicine and Surgery, Nashville, Tenn.; New Orleans Medical and Surgical Journal, New Orleans, La.; Obstetrical Journal, Philadelphia, Pa.; Ohio Medical Record, Columbus, O.; Oregon Medical Journal, Salem, Oregon; Pacific Medical and Surgical Journal, San Francisco, Cal.; Philadelphia Medical Times, Philadelphia, Pa., Physician and Pharmacist, New York; Quarterly Journal, Hartford, Conn.; Richmond and Louisville Medical Journal, Louisville, Ky.; Saint Louis Clinical Record, Saint Louis, Mo.; Saint Louis Medical and Surgical Journal, Saint Louis, Mo.; Sanitarian, New York; Texas Medical Journal, Galveston, Texas; The Clinic, Cincinnati, O.; Toledo Medical and Surgical Journal, Toledo, O.; Virginia Medical Monthly, Richmond, Va.; Western Lancet, San Francisco, Cal.

